# Temporal Dietary Patterns and Frailty in Korean Older Adults: Evening-Skewed and Morning–Evening Eating Patterns Associated with Frailty Risk

**DOI:** 10.3390/nu18040701

**Published:** 2026-02-22

**Authors:** Han Byul Jang, Sarang Jeong, Min-Ju Kim, Hyun-Joung Lim, Kyung Eun Lee

**Affiliations:** 1 Division of Cardiovascular Disease Research, Department of Chronic Disease Convergence Research, Korea National Institute of Health, Korea Disease Control and Prevention Agency, Cheongju 28159, Republic of Korea; 2 College of Pharmacy, Chungbuk National University, Cheongju 28160, Republic of Korea; 3 Industry-Academy Collaboration Foundation, Dongduk Women’s University, Seoul 02748, Republic of Korea

**Keywords:** temporal dietary pattern, frailty, chrono-nutrition, circadian rhythm, energy intake, diet quality

## Abstract

Background: Meal timing has emerged as a potential determinant of healthy aging; however, evidence linking temporal dietary patterns (TDPs) with frailty remains limited. We aimed to identify distinct TDPs among older adults and examine their associations with frailty and its components. Methods: In this cross-sectional study, 4184 adults aged ≥ 65 years from the Korea National Health and Nutrition Examination Survey (2016–2018) were analyzed. Temporal energy-intake trajectories from 24 h recalls were clustered using dynamic time warping-based kernel k-means. Frailty was defined using a modified Fried phenotype, and diet quality was assessed employing the Healthy Eating Index. Survey-weighted logistic regression and mediation analyses were performed. Results: Five distinct patterns were identified as follows: balanced (*n* = 1665, 38.8%), steady (*n* = 735, 17.8%), midday (*n* = 737, 18.0%), evening (*n* = 627, 15.2%), and morning–evening (*n* = 420, 10.2%). Among these, the evening-skewed (characterized by a disproportionate concentration of energy intake in the late evening; adjusted odds ratio [OR] = 1.48, 95% confidence interval [CI] = 1.03–2.10) and morning–evening (characterized by higher energy intake in both the morning and evening; OR = 1.43, 95% CI = 1.01–2.03) patterns were associated with higher frailty risk than the balanced pattern. Mediation analysis showed that higher total energy intake had a protective role in the evening-skewed pattern; however, this benefit was offset by the adverse impact of late-night eating, resulting in increased frailty risk. In the morning–evening pattern, both a direct association with frailty and an indirect pathway mediated by lower energy intake and poorer diet quality contributed to the increased frailty risk. Conclusions: Older adults with evening-skewed or morning–evening TDPs had greater frailty risk than those with balanced eating patterns. Optimizing meal timing—by reducing late-day energy loading and ensuring adequate overall intake and dietary quality—may represent a feasible chrono-nutrition strategy for frailty prevention.

## 1. Introduction

Frailty is a geriatric clinical syndrome characterized by reduced physiological reserve and increased vulnerability to stressors, leading to adverse outcomes, including disability, hospitalization, and mortality [1,2]. Identifying modifiable risk factors has become a public health priority since frailty is considered preventable and potentially reversible. Among these, nutrition has consistently emerged as a central determinant, with inadequate dietary intake and poor diet quality linked to sarcopenia, systemic inflammation, impaired immune function, and increased frailty risk [3,4].

Most previous research has emphasized what older adults eat—dietary quantity and quality—rather than when they eat. However, growing evidence suggests that the timing of food intake, within the broader concept of chrono-nutrition, plays an important role in health. Chrono-nutrition encompasses the timing, frequency, and duration of eating episodes, as well as their alignment with circadian rhythms [5]. Irregular eating schedules, prolonged eating windows, breakfast skipping, and late-night energy intake have been associated with adverse metabolic outcomes, including obesity, insulin resistance, and cardiovascular risk [6,7,8]. Moreover, misalignment between meal timing and circadian biology may accelerate aging processes [5,9].

Few studies have linked meal-timing behaviors to frailty. Evidence from Japanese cohorts reveals that breakfast skipping and having a low meal frequency are associated with a higher prevalence of prefrailty and frailty [10,11]. Building on these findings, a recent European multinational cohort showed that habitual meal skipping in earlier life stages was associated with an increased likelihood of developing physical frailty in later life [12]. Moreover, uneven protein distribution across meals has been associated with impaired muscle function in older adults with frailty, as muscle health is a key physiological determinant of frailty [13]. Similarly, a previous study reported that earlier timing of the first meal, later last intake time, and longer eating windows were positively correlated with muscle mass and grip strength [14].

Despite these emerging findings, most existing studies have examined isolated aspects of meal timing—such as skipping behaviors, meal frequency, eating window, or overall diet quality—without considering the comprehensive temporal distribution of energy intake across the day. Given the inherently time-structured nature of dietary intake, a trajectory-based approach that captures the full within-day energy distribution may provide a more integrative characterization of daily eating patterns. Dynamic time warping (DTW)–based clustering enables the identification of temporal dietary patterns by accommodating individual variability in meal timing while preserving similarities in daily energy distribution profiles. To address this gap, the present study applied DTW-based clustering to identify data-driven temporal dietary patterns (TDPs) using nationally representative data from the Korea National Health and Nutrition Examination Survey (KNHANES). We subsequently examined the associations between these TDPs and frailty status and further investigated whether total energy intake and overall diet quality mediated these relationships. We hypothesized that distinct TDPs would show differential associations with frailty among older adults, beyond the effects of total energy intake and overall diet quality. By integrating a chrono-nutrition perspective into frailty research, this study seeks to determine whether the quantity, quality, and timing of dietary intake independently contributes to frailty among older adults.

## 2. Materials and Methods

### 2.1. Study Population

This study used data from the seventh KNHANES (2016–2018) [15]. The KNHANES is a nationwide, cross-sectional survey that provides nationally representative data on health status, nutritional intake, and socioeconomic indicators among the Korean population [16]. The survey is conducted annually by the Korea Disease Control and Prevention Agency (KDCA) using a stratified, multistage, probability-cluster sampling design based on geographic area, housing type, and household unit. A total of 192 primary sampling units (survey districts) were selected in the 2016 survey cycle, with 23 households sampled from each unit. All household members aged ≥1 year who met the eligibility criteria were invited to participate.

A total of 24,269 individuals participated in the 2016–2018 health examination survey. In accordance with the definition of older adults used in Korean public health policy and national surveys, the study population was restricted to individuals aged ≥65 years (*n* = 4956). After excluding participants without 24 h dietary recall data, 4471 individuals remained. Participants with implausible energy intake (<500 or >5000 kcal/day for females; <500 or >6000 kcal/day for males) were excluded, yielding 4402 participants. The exclusion of those with insufficient frailty information resulted in a final analytic sample of 4184 older adults (Figure S1).

### 2.2. Assessment of Sociodemographic and Health-Related Variables

Sociodemographic variables included age, sex, education attainment, household income, economic activity, and living arrangement. Age was categorized as 65–74 or ≥75 years, while educational attainment was classified as middle school or less, high school, or college or higher. Household income was classified into high, middle-high, middle-low, and low quartiles. Economic activity was defined as current employment (yes/no), while living arrangement was categorized as living alone or living with others.

Health-related characteristics included current smoking, current alcohol drinking, sleep duration, chewing difficulty, and meal frequency. Sleep duration referred to the average weekday sleep time (h/day) and was categorized as ≤6, 6–8, or ≥9 h. Chewing difficulty was defined as responding “very uncomfortable” or “uncomfortable” to the chewing ability question. Physical activity level was assessed using the Global Physical Activity Questionnaire (GPAQ) and categorized as low (<600 Metabolic Equivalent of Task [MET]-min/week), moderate (600–2999 MET-min/week), or high (≥3000 MET-min/week) based on total MET-minutes per week, following World Health Organization criteria. Meal frequency—assessed based on self-reported breakfast, lunch, and dinner consumption over the past year—was categorized as <3 or ≥3 days/week.

### 2.3. Assessment of Health-Related Quality of Life

Health-related quality of life was assessed using the EuroQol 5-dimension questionnaire (EQ-5D), a standardized instrument widely used to assess functional health status in population-based studies. The EQ-5D evaluates five domains—mobility, self-care, usual activities, pain/discomfort, and anxiety/depression—each with three response levels (no, some, and extreme problems). Responses were converted into a single index score using the Korean population-based EQ-5D-5L valuation set [17]. Higher scores indicate better health-related quality of life.

### 2.4. Assessment of Frailty

Frailty was assessed using a modified Fried frailty phenotype [1], which includes the following five components: unintentional weight loss, weakness, exhaustion, slowness, and low physical activity. Unintentional weight loss was defined as a self-reported loss of ≥3 kg during the past year. Weakness was assessed using maximal grip strength measured across up to three trials, with cutoffs of <28 and <18 kg for men and women, respectively, according to the 2019 Asian Working Group for Sarcopenia consensus [18]. Exhaustion was defined as self-reporting having “felt very much stressed,” reflecting subjective exhaustion. Slowness and low physical activity were operationalized using items from the EQ-5D index, which has been widely applied to capture functional limitations in population-based studies. Specifically, slowness was defined as reporting some or extreme problems in the mobility domain, while low physical activity was defined as reporting some or extreme problems in the usual activities domain. Participants meeting three or more of the five criteria were classified as frail.

### 2.5. Dietary Assessment

Dietary intake was assessed using a single 24 h dietary recall from the KNHANES, which collected detailed information on the types, amount, and timing of all foods consumed at breakfast, lunch, and dinner, as well as snacks. Reported foods were converted to energy and nutrient intakes using the Korean Food Composition Table provided by the Rural Development Administration [15].

The Korean Healthy Eating Index (HEI) was applied to evaluate overall dietary quality [19]. Specifically, the HEI was derived from the 24 h recall and comprised three components as follows: (i) adequacy (e.g., breakfast consumption, whole grains, fruits, vegetables excluding pickled vegetables, protein foods, and dairy products), (ii) moderation (e.g., sodium, saturated fat, and added sugars), and (iii) balance (e.g., carbohydrate and fat energy ratios and energy adequacy). Higher HEI scores indicated better dietary quality.

### 2.6. Temporal Dietary Pattern (TDP) Identification

TDPs, representing daily energy distribution profiles, were identified using kernel k-means clustering with DTW distance [20], which aligns time series to more effectively capture similarities in temporal intake trends than Euclidean distance [21,22]. Hourly energy intake profiles were reconstructed using 24 h dietary recall data. Information on meal timing and meal type (breakfast, lunch, dinner, and snacks) was available. To improve comparability across individuals and to focus on the relative temporal distribution of daily energy intake, the three main meals were mapped to predefined standard time windows (breakfast: 06:00–08:00; lunch: 11:00–13:00; dinner: 17:00–19:00). Snack consumption, which exhibits greater temporal variability, was assigned based on the reported actual time of intake. Hourly energy intake was then aggregated and expressed as a proportion of each individual’s total daily energy intake. Accordingly, clustering analyses focused on identifying patterns of energy distribution across the day rather than differences in absolute energy intake. The optimal number of clusters (K) was determined using a rank aggregation consensus approach that integrates results from multiple internal validity indices (Figure S2). Candidate solutions (K = 2–7) were evaluated using the Silhouette [23], Dunn [24], Davies–Bouldin [25], Modified Davies–Bouldin [26], and Calinski–Harabasz [27] indices. Each index ranked the candidate solutions and these rankings were aggregated using a Borda count-based consensus method, following established frameworks for rank aggregation in clustering and decision analysis [28]. The solution with the lowest aggregated rank score was selected as the optimal K, providing a consensus-driven result robust across multiple evaluation criteria.

To aid interpretation of the identified TDPs within a chrono-nutrition framework, several timing- and frequency-related dietary indicators were examined, including meal-specific energy and protein distribution, meal skipping, eating window, and eating midpoint [5,7,11,14]. Energy and protein intake at breakfast, lunch, and dinner was expressed as a proportion of total daily intake. Meal skipping for each meal was defined based on whether the meal was reported on the day preceding the dietary survey. The eating window was defined as the interval between the first and last eating occasions, while the eating midpoint was defined as the halfway point of this interval.

### 2.7. Statistical Analysis

Statistical analyses were performed using SAS version 9.4 (SAS Institute, Cary, NC, USA). Analyses incorporated strata, clusters, and sampling weights using survey procedures since KNHANES employs a complex sampling design. Continuous variables were summarized as weighted means with standard errors and compared using “PROC SURVEYREG.” Post hoc comparisons were performed using Scheffé’s method when overall differences were significant. Categorical variables are presented as frequencies and weighted percentages and were compared using the Rao–Scott χ^2^ test. Associations between TDPs and frailty were examined using multivariable survey-weighted logistic regression. Sensitivity analyses were additionally conducted to evaluate the robustness of the main findings, including models applying design-specific survey weights and models separately adjusting for total energy intake or HEI. Survey-weighted mediation analyses were conducted using the “svydesign” framework in R version 4.5.1 (R Foundation, Vienna, Austria) to assess whether total energy intake and HEI mediated the associations between dietary pattern clusters (Cluster 1 vs. 4 and 1 vs. 5) and frailty. Indirect, direct, and total effects were estimated using generalized linear models, and 95% percentile bootstrap confidence intervals (CIs; 5000 replications) were used for indirect effects. Statistical significance was defined as a two-sided *p* < 0.05.

### 2.8. Ethics Statement

The study protocol was approved by the Institutional Review Board of the Korean National Institute of Health (approval number: KDCA-2024-06-11-C-04). The analysis was based on secondary data obtained from the KNHANES (2016–2018). The dataset was fully anonymized prior to access, and no personally identifiable information was available to the investigators. Therefore, the requirement for informed consent was waived by the Institutional Review Board.

## 3. Results

### 3.1. Characteristics of TDP Clusters

**Figure 1:**
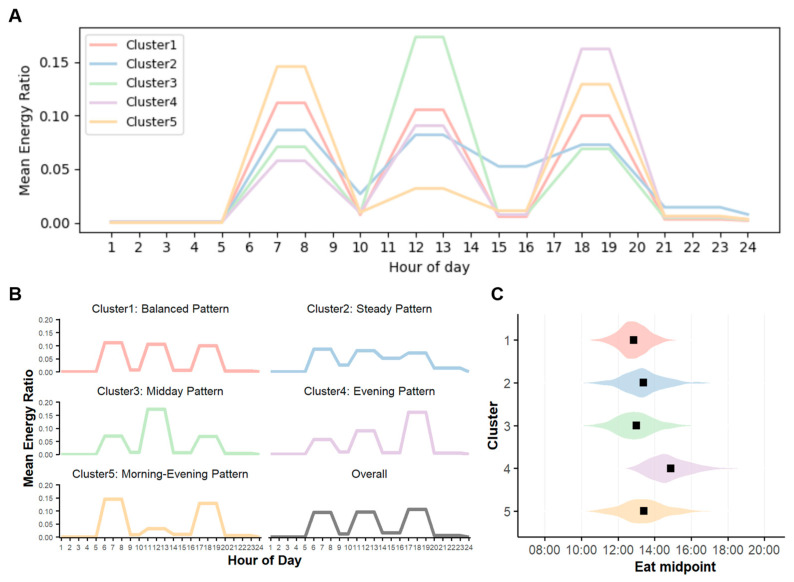
Temporal dietary pattern (TDP) clusters among older adults. (**A**) The 24 h distribution of energy intake across five clusters identified using dynamic time warping-based clustering. (**B**) Cluster 1 represents a balanced pattern with comparable energy contributions from breakfast, lunch, and dinner. Cluster 2 represents a steady pattern characterized by consistent intake across three meals with an additional afternoon snack. Cluster 3 denotes a midday pattern with energy intake concentrated around lunch and lower intake at breakfast and dinner. Cluster 4 describes an evening pattern in which most energy is consumed in the evening with minimal intake in the morning. Cluster 5 denotes a morning–evening pattern with peaks at breakfast and dinner and lower intake at midday. (**C**) Distribution of eating midpoints across clusters illustrating distinct temporal centroids. Black squares represent the median eating midpoint within each cluster.

Kernel k-means clustering with DTW distance identified five distinct TDPs among older adults (Table 1 and Figure 1). Cluster 1 (balanced pattern) comprised the largest proportion of participants (38.8%). It was characterized by an even distribution of energy intake across breakfast, lunch, and dinner, with the lowest prevalence of meal skipping (<1.2%) and earliest intake midpoint (12:52 ± 0:01). Cluster 2 (steady pattern, 17.8%) featured three main meals and an afternoon snack, indicating relatively continuous intake throughout the day. This cluster showed a high proportion of daytime energy intake (40.1%), and the longest eating window (11:56 ± 0:07). Cluster 3 (midday pattern, 18.0%) had the majority of energy intake at lunch (54.5%), with relatively low intake during breakfast and dinner. It had the second-earliest intake midpoint (13:02 ± 0:03), reflecting a daytime-oriented eating pattern. Cluster 4 (evening pattern, 15.2%) was defined by the highest proportion of energy intake (51.4%) in the evening, along with the latest intake midpoint (14:51 ± 0:04). Moreover, it had the highest prevalence of breakfast skipping (17.9%). Cluster 5 (morning–evening pattern, 10.2%) displayed a bimodal intake distribution, with energy intake peaks at breakfast (45.5%) and dinner (41.6%) and minimal intake at lunch. More than half of participants in this cluster reported lunch skipping (51.9%), and this group had the shortest eating window (10:58 ± 0:08).

### 3.2. Sociodemographic and Health-Related Characteristics

Significant differences were observed in sociodemographic and health-related characteristics across the five TDP clusters (Table 2 and Figure S3). With respect to sociodemographic characteristics, mean age was highest in Clusters 1 and 5 and lowest in Cluster 4 (*p* < 0.0001). Sex distribution differed across clusters, with a predominance of females in Cluster 5 (67.5%) and a higher proportion of males in Cluster 4 (49.4%). Lower educational attainment and household income were more frequently observed in Clusters 1 and 5 (both *p* < 0.001). Current employment was most common in Cluster 4 (39.8%) and least common in Cluster 5 (24.8%) (*p* = 0.0012), while living alone was most prevalent in Cluster 5 (32.4%) (*p* = 0.003). Regarding lifestyle factors, current alcohol consumption was highest in Cluster 4 (50.1%) and lowest in Cluster 5 (25.5%), respectively (*p* < 0.0001). Meal skipping habit, defined as consuming a given meal on fewer than three days per week during the past year, differed by cluster: breakfast skipping was most frequent in Cluster 4 (9.3%), lunch skipping in Cluster 5 (27.0%), and dinner skipping in Cluster 2 (1.8%) (all *p* < 0.0001). Health-related quality of life, assessed by the EQ-5D index, was lowest in Cluster 5 (*p* = 0.0447). However, no significant differences were observed across clusters for sleep duration, chewing difficulty, or physical activity.

Compared with participants included in the final analytic sample, excluded participants were less likely to live alone, reported higher alcohol consumption, and had a higher prevalence of dinner skipping (Table S1).

### 3.3. Nutrient Intake and Diet Quality Across Temporal Dietary Pattern Clusters

**Figure 2:**
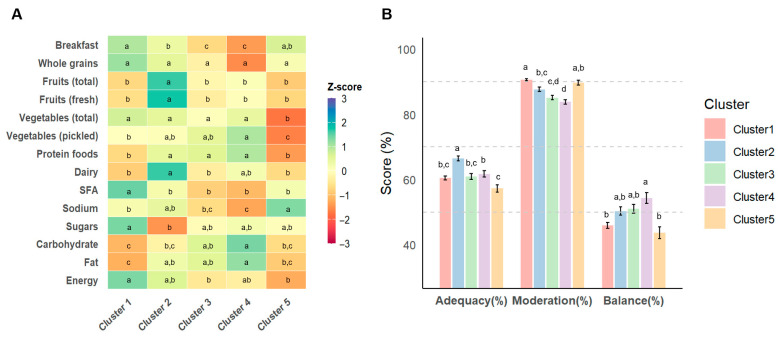
Comparison of the Healthy Eating Index (HEI) across temporal dietary pattern (TDP) clusters. (**A**) Heatmap showing standardized z-scores of individual HEI components across the five clusters. (**B**) Bar plots display mean percentage scores (±SE) of the three HEI subcategories by cluster: adequacy (breakfast, whole grains, fruits, vegetables, protein foods, and dairy), moderation (SFA, sodium, and sugars), and balance (carbohydrate, fat, and energy). Distinct superscript letters denote significant differences between clusters (*p* < 0.05, Scheffé test). SFA: saturated fat; TDP: temporal dietary pattern; HEI: Healthy Eating Index; SE: standard error.

Table S2 and Figure 2 summarize nutrient intake and diet quality across TDP clusters. Total energy and protein intake differed significantly across TDP clusters (Table S2), with the highest intake observed in Cluster 4 and the lowest in Cluster 5 (*p* < 0.0001). Diet quality, assessed by the HEI, also significantly differed across clusters (*p* < 0.0001). Cluster 2 had the highest overall HEI score, driven by superior adequacy and balance sub-scores. In contrast, Cluster 5 had the lowest overall HEI, reflecting consistently poorer intake of fruits, vegetables, protein sources, and dairy, as well as imbalanced macronutrient distribution. Breakfast adequacy was lowest in Cluster 4, consistent with its higher prevalence of breakfast skipping. Regarding moderation sub-scores, Cluster 1 exhibited the most favorable profile, whereas Cluster 4 had the lowest scores due to higher intakes of saturated fats and sodium.

### 3.4. Associations Between TDPs and Frailty

Table 3 presents the associations between TDP clusters and frailty. All analyses incorporated the unified health survey and nutrition weights to ensure comparability across models. In Model 2, which adjusted for sociodemographic and lifestyle factors (age, sex, household income, education, economic activity, living arrangement, and alcohol consumption), frailty risk was higher in Clusters 4 (odds ratio [OR] = 1.44; 95% CI: 1.01–2.05) and 5 (OR = 1.60; 95% CI: 1.14–2.25) than in Cluster 1. These associations persisted after additional adjustment for total energy intake and HEI (Cluster 4: OR = 1.48; 95% CI: 1.03–2.10; Cluster 5: OR = 1.43; 95% CI: 1.01–2.03). No significant associations were observed for Clusters 2 or 3. Sensitivity analyses, including models using design-specific weights (Models 1 and 2) and models separately adjusting for total energy intake (Model 4) or HEI (Model 5), yielded results consistent with the main analysis (Table S3).

### 3.5. Associations Between TDP Clusters, Frailty Components, and EQ-5D Dimensions

**Figure 3:**
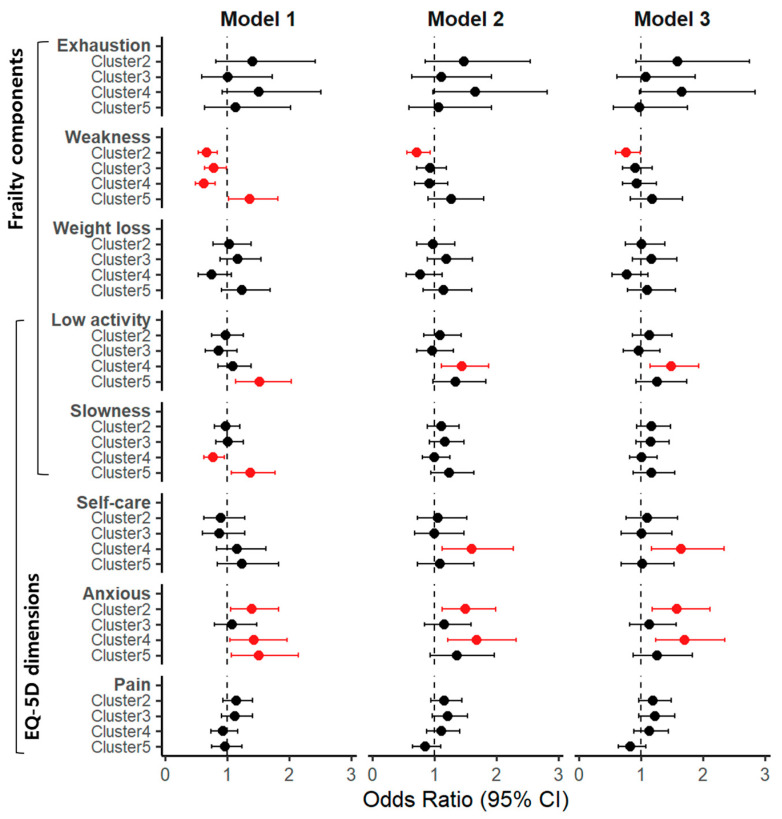
Logistic regression-derived odds ratios for frailty components and EQ-5D dimensions. Cluster 1 (Balanced pattern) served as the reference group. Unified survey weights were applied to harmonize estimates across health interview, examination, and nutrition survey components. Model 1 was unadjusted; Model 2 was adjusted for age, sex, household income, education, economic activity, living arrangement, and alcohol consumption; Model 3 was additionally adjusted for total energy intake and Healthy Eating Index (HEI) scores. Red highlights statistically significant results (*p* < 0.05). CI: confidence interval; EQ-5D: EuroQol 5-dimension questionnaire.

Figure 3 summarizes the associations between TDP clusters and individual frailty components and EQ-5D dimensions. For frailty components, weakness was less common in Clusters 2, 3, and 4 compared with Cluster 1, although statistical significance across all adjusted models was observed only for Cluster 2. Cluster 5 showed a higher risk of weakness in unadjusted analyses, which was attenuated after adjustment. Unintentional weight loss did not differ significantly across clusters. Low physical activity was consistently more frequent in Cluster 4, while exhaustion showed a nonsignificant tendency toward higher risk. Slowness differed only in crude models, with lower and higher risks observed in Clusters 4 and 5, respectively. For EQ-5D dimensions, anxiety was consistently more prevalent in Clusters 2 and 4 across all models, and self-care limitations were more frequent in Cluster 4. No significant differences were observed for pain/discomfort. Although sensitivity analyses revealed some variation in statistical significance, overall patterns remained consistent with the primary findings (Figure S4).

### 3.6. Mediation Analysis of Energy Intake and Diet Quality in Relation to Frailty

**Figure 4:**
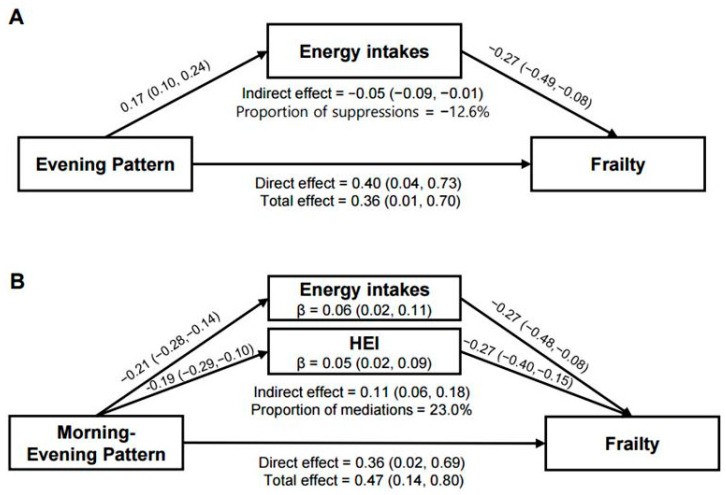
Mediation analysis of indirect and direct effects of temporal dietary patterns (TDPs) on frailty. (**A**) Evening-skewed and (**B**) morning–evening patterns were evaluated using total energy intake and Healthy Eating Index (HEI) scores as parallel mediators. All models were adjusted for age, sex, household income, educational attainment, economic activity, living arrangement, and alcohol consumption.

Mediation analyses were performed for Clusters 4 and 5, which are the two clusters associated with higher frailty risk (Figure 4). All models were adjusted for sociodemographic and lifestyle factors. HEI was not associated with the exposure in Cluster 4, but was inversely associated with frailty; therefore, it was treated as an adjustment covariate rather than a mediator (Figure S5). In this HEI-adjusted model, Cluster 4 was associated with higher total energy intake (β = 0.17; 95% CI: 0.10–0.24), which was inversely associated with frailty (β = −0.27; 95% CI: −0.49 to −0.08). This pathway produced a suppressive indirect effect (indirect effect = −0.05; 95% CI: −0.09 to −0.01; 12.6% mediated), while the direct effect of the evening pattern remained predominant. For Cluster 5, total energy intake was lower (β = −0.21; 95% CI: −0.28 to −0.14) and HEI was reduced (β = −0.19; 95% CI: −0.29 to −0.10). Both indicators were associated with higher frailty risk (energy intake: β = −0.27; 95% CI: −0.48 to −0.08; HEI: β = −0.27; 95% CI: −0.40 to −0.15). The combined indirect effect was significant (indirect effect = 0.11; 95% CI: 0.06–0.18), accounting for 23.0% of the total effect, whereas the direct effect also remained significant (β = 0.36; 95% CI: 0.02–0.69).

## 4. Discussion

Five distinct TDPs were identified using kernel k-means clustering with DTW distance in this study of older Korean adults. The evening-skewed and morning–evening patterns were associated with higher frailty risk than the Balanced pattern. Mediation analyses indicated that the mechanisms underlying these associations differed by pattern. Higher total energy intake appeared protective in the evening-skewed pattern but was counteracted by the adverse impact of late eating; however, lower energy intake and poorer diet quality partially mediated the frailty association in the morning–evening pattern. These findings highlight the importance of both balanced meal timing and nutritional adequacy in strategies to prevent frailty in aging populations.

Previous studies on diet and frailty have largely focused on dietary quality and composition [4,29,30,31,32], paying limited attention to the temporal distribution of intake. However, emerging evidence suggests that meal timing and regularity may also be relevant to frailty-related vulnerability. In a German sample of community-dwelling adults aged ≥75 years, individuals with frailty exhibited a more unfavorable within-day distribution of intake—characterized by lower morning intake and a more uneven distribution of protein intake across meals—even when total intake was comparable across frailty groups [33]. Consistent with this pattern, an evening-concentrated distribution of energy intake has been associated with adverse cardiometabolic outcomes that overlap with established frailty pathways; specifically, higher energy intake at dinner predicted incident metabolic syndrome in older adults [34]. Further supporting the relevance of late eating in aging, a 20-year longitudinal study showed that individuals following trajectories characterized by progressively later meal timing and a later eating midpoint were more likely to experience fatigue and multimorbidity, and that later breakfast timing was associated with higher mortality risk [35]. In this context, our findings provide complementary evidence by demonstrating that an evening-skewed distribution of intake is associated with higher frailty risk in older adults, independent of total energy intake and diet quality, suggesting that late and evening-concentrated eating patterns may represent a potentially modifiable marker along the pathway to functional vulnerability and long-term health decline.

Several biological and behavioral mechanisms may explain the higher frailty risk associated with the evening-skewed pattern. First, delayed and concentrated energy intake may contribute to circadian misalignment. Core metabolic processes, including insulin sensitivity, glucose regulation, lipid oxidation, and mitochondrial function, follow diurnal rhythms and operate most efficiently earlier in the day [5,6,36,37,38,39]. Consuming a large proportion of daily energy intake in the evening, when metabolic capacity declines, may impose greater physiological strain, promote oxidative stress, and impair nocturnal recovery [14,37,39]. Second, late-day energy loading is associated with hormonal alterations, such as a reduced morning cortisol awakening response, blunted melatonin rhythm, and disrupted leptin–ghrelin balance [5,9,40,41]. These disturbances may contribute to fatigue, sleep fragmentation, and reduced physical capacity, which are core elements of the frailty cycle. In line with this, recent evidence in older adults suggests that a later eating midpoint is associated with greater psychological vulnerability, including depressive symptoms [35]. Consistently, the evening-skewed pattern in the present study was characterized by higher levels of fatigue, activity limitations, and anxiety, suggesting that such metabolic and hormonal disruptions may translate into functional vulnerability. Previous studies further suggest that key metabolic and hormonal regulatory systems differ by sex in terms of baseline levels and regulatory dynamics [42,43]. However, whether these sex-related differences lead to differential susceptibility to external behavioral factors, such as meal timing, remains unclear. In the sex-stratified analyses in the current study, the association between an evening-skewed dietary pattern and frailty was statistically significant in men, while a similar direction of association was observed in women, with no evidence of a significant sex-by-pattern interaction (Table S4). Together, these findings suggest that late-day energy intake may be relevant to frailty risk in both sexes, although the strength of the observed associations may differ according to sex-specific physiological or hormonal contexts.

The morning–evening pattern was characterized by bimodal peaks in energy intake at breakfast and dinner, a shortened eating window, and a markedly higher prevalence of lunch skipping, leading to lower total energy intake and poorer diet quality, which partially explains its association with increased frailty risk. A substantial body of observational studies and meta-analyses has consistently shown that low energy intake and undernutrition are key contributors to frailty development and progression in older adults [4,29,30,31,32,44]. Although few studies have examined lunch skipping, prior evidence indicates that older adults who repeatedly skip meals are more likely to experience insufficient total energy and protein intake, micronutrient deficiencies, and undernutrition [45,46]. Moreover, consuming < 3 meals per day—particularly two meals or fewer—is significantly associated with a higher risk of frailty [10,11], which aligns with the increased frailty risk observed in the morning–evening pattern in our study.

Physiologically, repeated omission of lunch may reduce opportunities for protein and micronutrient intake during midday—a period critical for muscle protein synthesis—thereby limiting amino acid availability and impairing muscle maintenance [47,48]. Prolonged daytime fasting may also result in compensatory evening energy loading, increased glycemic variability, and worsened insulin resistance, contributing to frailty through chronic metabolic stress [12,49,50]. Beyond metabolic implications, lunch represents an important opportunity for social engagement in older adults, and its habitual omission may increase social isolation and susceptibility to social frailty [46,51]. Although our study did not directly measure social networks, the higher proportion of individuals living alone in the morning–evening group provides indirect support for this pathway’s relevance. These multidimensional pathways also provide a plausible explanation for the absence of elevated frailty risk in the midday-skewed pattern, in which intake was concentrated at lunch. However, lunch skipping per se was not independently associated with frailty in the present study (Table S5). Rather, our findings suggest that the association with frailty is better understood in the context of a broader dietary pattern characterized by unfavorable timing and intake distribution. In this context, the morning–evening pattern reflects overall daily eating behavior beyond an isolated meal-skipping behavior. Although none of the individual frailty components reached statistical significance, the elevated overall frailty risk suggests that small physiological, metabolic, and social deficits may act cumulatively to exceed the clinical threshold for frailty.

Interestingly, the steady pattern, characterized by frequent meals and snacks distributed across a relatively long eating window, was associated with a lower risk of weakness. Although a longer eating window may appear inconsistent with the purported benefits of time-restricted eating (TRE), most evidence supporting TRE is derived from younger, metabolically healthy adults with high physical activity levels [52,53]. Recent findings in older adults show that earlier first meals, later last meals, and longer eating windows are linked to greater muscle mass and better functional status, suggesting that optimal meal-timing patterns can shift with age [14]. A plausible explanation is that more frequent eating occasions facilitate a more even distribution of protein intake throughout the day, enhancing amino acid availability and supporting repeated stimulation of muscle protein synthesis, which is critical for preserving muscle mass in older adults [14,47,48]. These considerations align with the lower prevalence of weakness observed in the steady pattern in our study and suggest that optimal meal timing structures in older adults fundamentally differ from those beneficial in younger or more metabolically robust groups.

However, frequent eating is not inherently indicative of healthy behavior. Several studies suggest that psychological distress manifests as repeated or continuous eating episodes [54], which can provide transient relief but eventually lead to fatigue and impaired sleep quality [55,56]. Such eating patterns may also reflect emotional dysregulation and impulsive responding under negative affect (negative urgency) [54], which may increase vulnerability to emotional frailty [57]. Consistent with this interpretation, the steady pattern in our study was associated with a higher prevalence of anxiety symptoms. Therefore, this pattern may serve as an early indicator of emerging emotional vulnerability, even in the absence of noticeable physical decline.

This study has several strengths, including the use of nationally representative data, the application of DTW-based clustering to capture TDPs, the rank aggregation of multiple validity indices to identify the optimal cluster number, the use of survey-weighted mediation models to disentangle direct and indirect pathways, and the joint evaluation of frailty and quality-of-life measures to provide a multidimensional interpretation of findings. Importantly, this temporal pattern-based chrono-nutrition framework extends prior research by shifting the analytical focus from isolated meal-timing behaviors to integrated whole-day eating rhythms, an approach that has received limited attention in older adults, particularly in relation to frailty. Nonetheless, some limitations should be acknowledged. First, the cross-sectional design limits causal inference, and longitudinal studies are required to determine whether changes in TDPs precede or follow frailty progression. Second, dietary data were based on a single 24 h recall, which may not fully capture intra-individual variability; however, reported meal skipping frequency aligned with participants’ habitual patterns, supporting the validity of our findings. Third, although multiple covariates were accounted for, unmeasured factors such as sleep patterns, social relationships, or chronotype may have influenced the observed associations. Fourth, some TDP clusters had relatively small sample sizes, resulting in wide CIs; however, the direction of associations remained consistent across sensitivity analyses. Finally, replication in other settings and cultural contexts is required to establish external validity, as the clustering-derived TDPs may be population-specific. In addition, although the possibility of selection bias cannot be excluded, comparisons between included and excluded participants suggest that its impact is likely to be limited, and this should be taken into account when interpreting the findings.

## 5. Conclusions

This study showed that TDPs were differentially associated with frailty among older adults. Late eating was associated with increased frailty risk in the evening-skewed pattern despite adequate total energy intake; however, lower energy intake, poorer diet quality, and frequent lunch skipping contributed to frailty risk in the morning–evening pattern. These findings indicate that meal timing and the temporal balance of energy distribution are important considerations alongside total energy intake and diet quality. The results also support incorporating a chrono-nutrition framework into frailty prevention strategies by highlighting meal timing and energy distribution as potential intervention targets. Longitudinal and intervention studies are required to clarify causal pathways and determine the applicability of our findings in clinical and public health settings.

## Figures and Tables

**Table 1:** Characteristics of temporal dietary pattern clusters defined by a dynamic time warping method (*n* = 4184) ^1^.

Characteristics	Overall	Cluster 1	Cluster 2	Cluster 3	Cluster 4	Cluster 5
		(*n* = 1665, 38.8%)	(*n* = 735, 17.8%)	(*n* = 737, 18.0%)	(*n* = 627, 15.2%)	(*n* = 420, 10.2%)
Temporal patterns		Balanced Pattern	Steady Pattern	Midday Pattern	Evening Pattern	Morning–Evening Pattern
		Energy evenly distributed across breakfast, lunch, and dinner	Three meals plus afternoon snack, steady intake throughout the day	Major intake at lunch, low intake in the morning and dinner	Largest intake in the evening, little in the morning	Peak at breakfast and dinner, low intake at lunch
Energy/Protein intake distribution (%) ^2^						
Morning	30.7/30.4	35.1/34.6	31.4/32.3	22.7/21.2	18.9/18.5	45.5/45.2
Daytime	35.6/35.1	33.3/33.2	40.1/37.8	54.5/55.7	29.7/29.3	12.9/10.6
Evening	33.6/ 34.5	32.6/ 32.2	28.5/ 29.9	22.8/23.1	51.4/52.2	41.6/44.2
Meal Skipping (%)						
Breakfast	5.7	0.2	3.7	11.9	17.9	0.0
Lunch	7.4	1.2	5.2	0.7	4.0	51.9
Dinner	4.7	1.2	8.5	13.5	1.0	2.5
Eating window ^3^	11:33 ± 0:03	11:45 ± 0:03 ^a,b^	11:56 ± 0:07 ^a^	11:16 ± 0:08 ^b,c^	11:23 ± 0:07 ^b,c^	10:58 ± 0:08 ^c^
Eat midpoint ^3^	13:22 ± 0:02	12:52 ± 0:01 ^c^	13:23 ± 0:05 ^b^	13:02 ± 0:03 ^c^	14:51 ± 0:04 ^a^	13:33 ± 0:05 ^b^

^1^ The sample sizes (n) represent the number of observations in the data, whereas proportions (%) are weighted to account for the complex survey design. ^2^ Morning time was defined as 04:00–10:59, daytime as 11:00–16:59, and evening time as 17:00–03:59; Data are presented as survey-weighted means of the percentage contribution of each meal (Morning, Day, and Evening times) ^3^ Eating window and eating midpoint are expressed in hh ± mm format; Different superscription (^a,b,c^) denotes statistical differences between groups at 0.05 level (Scheffé test).

**Table 2:** Characteristics of participants according to temporal dietary pattern clusters.

Characteristics	Overall	Cluster 1	Cluster 2	Cluster 3	Cluster 4	Cluster 5	*p*-Value
Age	73.1 ± 0.13	73.6 ± 0.2 ^a^	72.7 ± 0.3 ^a,b^	72.9 ± 0.3 ^a^	71.6 ± 0.3 ^b^	74.1 ± 0.4 ^a^	<0.0001
65–74 y	2445 (56.7)	907 (52.4)	450 (60.6)	450 (58.3)	423 (67.9)	215 (47.1)	<0.0001
≥75 y	1739 (43.3)	758 (47.6)	285 (39.4)	287 (41.7)	204 (32.1)	205 (52.9)	
Sex							
Men	1823 (42.9)	757 (45.4)	300 (39.5)	311 (41.4)	299 (49.4)	156 (32.5)	0.0004
Women	2361 (57.1)	908 (54.6)	435 (60.5)	426 (58.6)	328 (50.6)	264 (67.5)	
Education							
<Middle school	2854 (69.6)	1203 (74.9)	497 (68.3)	471 (63.0)	391 (63.3)	292 (73.5)	0.0003
High school	710 (18.3)	232 (14.5)	134 (20.1)	149 (22.2)	128 (20.7)	67 (18.5)	
≥College graduate	458 (12.1)	149 (10.5)	82 (11.7)	97 (14.8)	84 (16.0)	46 (8.0)	
Household income							
Lowest (Q1)	2011 (46.9)	863 (50.0)	338 (43.0)	327 (45.2)	250 (41.2)	233 (53.5)	0.0002
Lower middle (Q2)	1122 (27.4)	443 (29.9)	201 (27.4)	198 (23.8)	187 (27.5)	93 (24.6)	
Upper middle (Q3)	607 (15.2)	220 (13.2)	115 (17.3)	130 (19.3)	95 (15.7)	47 (11.3)	
Highest (Q4)	423 (10.5)	128 (6.9)	77 (12.3)	81 (11.7)	92 (15.7)	45 (10.6)	
Current employment	1326 (31.7)	493 (28.8)	251 (34.3)	246 (32.5)	237 (39.8)	99 (24.8)	0.0012
Living alone	1001 (21.9)	400 (20.7)	174 (20.1)	160 (20.1)	143 (22.0)	124 (32.4)	0.0030
Current smoking	365 (8.4)	146 (8.7)	51 (5.8)	59 (7.1)	65 (12.1)	44 (9.0)	0.0306
Current drinking	1441 (34.7)	524 (32.9)	233 (30.4)	277 (35.3)	291 (50.1)	116 (25.5)	<0.0001
Sleep (h)	7.2 ± 0.04	7.2 ± 0.05	7.1 ± 0.08	7.2 ± 0.09	7.1 ± 0.09	7.3 ± 0.11	0.6166
<6 h	593 (14.8)	217 (15.4)	114 (16.4)	102 (13.0)	91 (15.0)	69 (16.1)	0.9055
6–8 h	2745 (68.5)	1075 (68.3)	490 (68.0)	504 (71.4)	420 (70.4)	256 (65.9)	
≥9 h	670 (16.7)	288 (16.3)	105 (15.6)	109 (15.6)	88 (14.7)	80 (18.1)	
Chewing difficulty	1783 (40.3)	696 (39.5)	319 (41.6)	305 (37.7)	266 (39.7)	197 (46.3)	0.2666
Physical activity							
Low	2830 (71.5)	1133 (71.4)	509 (71.6)	503 (70.8)	385 (69.0)	300 (76.1)	0.1542
Moderate	1073 (25.4)	397 (24.2)	187 (25.5)	193 (27.0)	196 (28.0)	100 (22.9)	
High	144 (3.1)	67 (4.4)	21 (2.9)	24 (2.2)	22 (3.0)	10 (1.0)	
Meal frequency (<3 days/week)							
Breakfast	160 (3.9)	13 (1.0)	23 (2.9)	55 (7.7)	63 (9.3)	6 (1.3)	<0.0001
Lunch	184 (4.7)	29 (1.5)	19 (4.1)	14 (1.7)	9 (1.8)	113 (27.0)	<0.0001
Dinner	42 (0.92)	9 (0.59)	13 (1.8)	11 (0.91)	4 (0.67)	5 (0.97)	<0.0001
EQ-5D	0.88 ± 0.00	0.89 ± 0.01	0.88 ± 0.01	0.89 ± 0.01	0.89 ± 0.01	0.85 ± 0.01	0.0447

Data are presented as survey-weighted means ± SE and weighted proportions (%) for continuous and categorical variables, respectively. Physical activity level was categorized as low, moderate, or high based on MET-minutes per week. Different superscription (^a,b^) denotes statistical differences between groups at 0.05 level (Scheffé test). EQ-5D: EuroQol 5-dimension questionnaire; MET, Metabolic Equivalent of Task; SE, standard error.

**Table 3:** Odds ratios (95% CI) for frailty by temporal dietary pattern cluster, using Cluster 1 (balanced pattern) as the reference.

Characteristics	Model 1		Model 2		Model 3	
Cluster 1	1	(Ref)	1	(Ref)	1	(Ref)
Cluster 2	1.05	(0.74–1.48)	1.24	(0.87–1.77)	1.36	(0.95–1.94)
Cluster 3	1.12	(0.81–1.55)	1.32	(0.94–1.84)	1.28	(0.92–1.80)
Cluster 4	1.00	(0.72–1.40)	**1.44**	**(1.01–2.05)**	**1.48**	**(1.03–2.10)**
Cluster 5	**1.76**	**(1.29–2.41)**	**1.60**	**(1.14–2.25)**	**1.43**	**(1.01–2.03)**

Models: Model 1: Unadjusted; Model 2: Adjusted for age, sex, household income, education, economic activity, living arrangement, and alcohol drinking; Model 3: Additionally adjusted for total energy intake and Healthy Eating Index scores. Statistical significance was considered at *p* < 0.05 and is shown in bold.

## Data Availability

The data presented in this study are available at https://knhanes.kdca.go.kr/knhanes/main.do (accessed on 10 February 2026).

## Supplementary Materials

The following supporting information can be downloaded at https://www.mdpi.com/article/10.3390/nu18040701/s1. Figure S1: Flow chart of the study design; Figure S2: Determination of the optimal number of clusters (K) using a rank-aggregation consensus approach; Figure S3: Forest plot showing odds ratios for sociodemographic and health-related characteristics across temporal dietary pattern clusters (reference = Cluster 1); Figure S4: Survey-weighted logistic regression analysis showing odds ratios (ORs) for individual frailty components and EQ-5D dimensions, using Cluster 1 (balanced pattern) as the reference group; Figure S5: Mediation analysis assessing the indirect and direct effects of temporal dietary patterns on frailty; Table S1: Comparison of baseline characteristics between included and excluded participants; Table S2: Nutrition intake across temporal dietary pattern clusters; Table S3: Survey-weighted logistic regression models with design-specific weights: Table S4: Sex-stratified associations between TDP and frailty, and tests for interaction; Table S5: Association between lunch skipping and frailty; Odds ratios (95% CI) for frailty by temporal dietary pattern cluster, using Cluster 1 (balanced pattern) as the reference group.

## Abbreviations

The following abbreviations are used in this manuscript:

DTW	Dynamic time warping
KNHANES	Korea National Health and Nutrition Examination Survey
TDP	Temporal Dietary Pattern
HEI	Healthy Eating Index
EQ-5D	EuroQol-5 Dimension questionnaire
TRE	Time-Restricted Eating
CI	Confidence interval
OR	Odds ratio
