# Supplementary material for: Temporal Dietary Patterns and Frailty in Korean Older Adults: Evening-Skewed and Morning–Evening Eating Patterns Associated with Frailty Risk
Source: Nutrients. 2026 Feb 22;18(4):701. doi: 10.3390/nu18040701 (PMC12943149; doi:10.3390/nu18040701)
Supplement: Supplementary file 1 [file nutrients-18-00701-s001.zip › nutrients-4102417-supplementary.pdf]

Supplementary Tables

Table S1. Comparison of baseline characteristics between included and excluded participants

Characteristics	Included		Excluded		P-value
Temporal patterns					
Age	73.1	± 0.13	72.7	± 0.31	0.2774
65-74y	2445	(56.7)	460	(57.2)	0.8759
≥75y	1739	(43.3)	312	(42.8)	
Sex					
Men	1823	(42.9)	312	(40.8)	0.5087
Women	2361	(57.1)	460	(59.2)	
Education					
< Middle school	2854	(69.6)	348	(72.0)	0.7682
High school	710	(18.3)	81	(16.7)	
≥ College graduate	458	(12.1)	54	(11.2)	
Household income					
Lowest	2011	(46.9)	366	(51.5)	0.6384
Lower middle	1122	(27.4)	218	(24.5)	
Upper middle	607	(15.2)	107	(14.0)	
Highest	423	(10.5)	66	(10.1)	
Current employment	1326	(31.7)	164	(36.4)	0.1802
Living alone	1001	(21.9)	137	(16.3)	0.0167
Current smoking	365	(8.4)	68	(12.8)	0.0233
Current drinking	1441	(34.7)	177	(38.1)	0.3237
Sleep (min)					
Sleep (hr)	7.2	± 0.04	7.2	± 0.10	0.7441
< 6 hr	593	(15.2)	71	(14.3)	0.0994
6-8 hr	2745	(68.9)	342	(74.6)	
≥ 9 hr	670	(16.0)	68	(11.0)	
Chewing difficulty	1783	(40.3)	224	(41.7)	0.7046
Physical activity					
Low (600<MET)	2830	(71.5)	338	(72.2)	0.584
Moderate (600-2,999 MET)	1073	(25.4)	132	(23.3)	0.5124
High (≥3,000 MET)	144	(3.1)	18	(4.4)	
Meal frequency (<3 days/week)					
Breakfast	160	(3.9)	25	(7.5)	0.104
Lunch	184	(4.7)	13	(6.4)	0.5124
Dinner	42	(0.92)	6	(3.6)	0.0318
EQ-5D	0.88	± 0.00	0.90	± 0.01	0.2945

Data are presented as survey-weighted means ± SE.

EQ-5D: EuroQol 5-dimension questionnaire; **SE:** standard error

Table S2. Nutrition intake across temporal dietary pattern clusters

Characteristics	Overall	Cluster 1	Cluster 2	Cluster 3	Cluster 4	Cluster 5	P-value
Energy & macronutrients							
Total energy intake (kcal)	1655.6 ± 14.7	1595.6 ± 16.8^{b}	1771.8 ± 30.8^{a}	1706.2 ± 39.4^{ab}	1800.7 ± 33.4^{a}	1365.2 ± 34.5^{c}	<.0001
Carbohydrates (g)	287.6 ± 2.5	290.4 ± 3.0^{b}	314 ± 5.9^{a}	285.7 ± 6.4^{b}	286.2 ± 5.5^{b}	236.5 ± 5.3^{c}	<.0001
Protein (g)	54.6 ± 0.6	52.3 ± 0.7^{c}	55.4 ± 1.1^{bc}	58.4 ± 1.5^{ab}	60.9 ± 1.5^{a}	45.9 ± 1.6^{d}	<.0001
Fat (g)	26.6 ± 0.4	22.2 ± 0.5^{b}	29 ± 1.0^{a}	30.2 ± 1.2^{a}	33.1 ± 1.1^{a}	22.9 ± 1.3^{b}	<.0001
Total Healthy Eating Index	67.5 ± 0.3	67.3 ± 0.4^{b}	70.4 ± 0.5^{a}	66.7 ± 0.6^{a}	67.2 ± 0.6^{b}	65 ± 0.7^{b}	<.0001
Adequacy items	33.8 ± 0.3	33.3 ± 0.3^{bc}	36.6 ± 0.4^{a}	33.5 ± 0.5^{bc}	33.9 ± 0.5^{b}	31.5 ± 0.6^{c}	<.0001
Breakfast	9.5 ± 0.0	9.9 ± 0.0^{a}	9.6 ± 0.1^{b}	9.1 ± 0.1^{c}	8.8 ± 0.1^{c}	9.7 ± 0.1^{ab}	<.0001
Whole grain	2.6 ± 0.0	2.8 ± 0.1	2.7 ± 0.1	2.5 ± 0.1	2.3 ± 0.1	2.6 ± 0.1	0.0262
Fruits(any type)	2.8 ± 0.1	2.5 ± 0.1^{b}	3.4 ± 0.1^{a}	2.7 ± 0.1^{b}	2.8 ± 0.1^{b}	2.5 ± 0.1^{b}	<.0001
Fruits(fresh only)	2.8 ± 0.1	2.7 ± 0.1^{b}	3.4 ± 0.1^{a}	2.8 ± 0.1^{b}	2.9 ± 0.1^{b}	2.6 ± 0.2^{b}	<.0001
Vegetables(any type)	3.6 ± 0.0	3.7 ± 0.0^{a}	3.7 ± 0.1^{a}	3.6 ± 0.1^{a}	3.7 ± 0.1^{a}	3.1 ± 0.1^{b}	<.0001
Vegetables(excluding pickled)	3.3 ± 0.0	3.3 ± 0.1^{b}	3.3 ± 0.1^{ab}	3.4 ± 0.1^{ab}	3.6 ± 0.1^{a}	2.9 ± 0.1^{c}	<.0001
Protein sources	6.6 ± 0.1	6.2 ± 0.1^{b}	6.9 ± 0.1^{a}	7 ± 0.2^{a}	7.2 ± 0.2^{a}	5.8 ± 0.2^{b}	<.0001
Dairy products	2.6 ± 0.1	2.2 ± 0.1^{b}	3.5 ± 0.2^{a}	2.4 ± 0.2^{b}	2.7 ± 0.2^{ab}	2.2 ± 0.2^{b}	<.0001
Moderation items	26.4 ± 0.1	27.2 ± 0.1^{a}	26.3 ± 0.2^{bc}	25.5 ± 0.2^{cd}	25.1 ± 0.2^{d}	26.9 ± 0.2^{ab}	<.0001
Satuated fat(%E)	9.1 ± 0.0	9.6 ± 0.0^{a}	9 ± 0.1^{b}	8.6 ± 0.1^{b}	8.4 ± 0.1^{b}	9 ± 0.1^{b}	<.0001
Sodium	7.8 ± 0.1	7.9 ± 0.1^{b}	8.1 ± 0.1^{ab}	7.5 ± 0.1^{bc}	7.2 ± 0.2^{c}	8.5 ± 0.2^{a}	<.0001
Sugar(%E)	9.5 ± 0.0	9.7 ± 0.0^{a}	9.2 ± 0.1^{b}	9.4 ± 0.1^{ab}	9.5 ± 0.1^{ab}	9.4 ± 0.1^{ab}	0.0002
Balance of energy items	7.3 ± 0.1	6.9 ± 0.1^{b}	7.5 ± 0.2^{ab}	7.7 ± 0.2^{ab}	8.2 ± 0.2^{a}	6.5 ± 0.3^{b}	<.0001
Carbohydrates(%E)	1.7 ± 0.0	1.4 ± 0.1^{c}	1.7 ± 0.1^{bc}	1.9 ± 0.1^{ab}	2.2 ± 0.1^{a}	1.5 ± 0.1^{bc}	<.0001

Fat(%E)	2.5	± 0.0	2.1	± 0.1[c]	2.7	± 0.1[ab]	2.7	± 0.1[ab]	3.0	± 0.1[a]	2.2	± 0.1[bc]	<.0001
Energy adequacy	3.2	± 0.0	3.4	± 0.1[a]	3.2	± 0.1[ab]	3.0	± 0.1[b]	3.1	± 0.1[ab]	2.8	± 0.1[b]	<.0001

Data are presented as survey-weighted means ± SE. Different superscript letters denote statistically significant differences between clusters ($p < 0.05$, Scheffé test).

SE: standard error

Table S3. Survey-weighted logistic regression models with design-specific weights: odds ratios (95% CI) for frailty by temporal dietary pattern cluster, using Cluster 1 (balanced pattern) as the reference group.

	Model 1		Model 2		Model 3		Model 4		Model 5	
Cluster 1	1	(Ref)	1	(Ref)	1	(Ref)	1	(Ref)	1	(Ref)
Cluster 2	1.14	(0.73–1.77)	1.33	(0.84–2.11)	1.36	(0.95–1.94)	1.30	(0.91–1.85)	1.32	(0.93–1.89)
Cluster 3	1.32	(0.90–1.93)	**1.53**	**(1.04–2.27)**	1.28	(0.92–1.80)	1.33	(0.95–1.86)	1.27	(0.90–1.77)
Cluster 4	1.03	(0.66–1.59)	1.57	(0.98–2.51)	**1.48**	**(1.03–2.10)**	**1.50**	**(1.06–2.14)**	**1.43**	**(1.00–2.04)**
Cluster 5	**2.15**	**(1.48–3.12)**	**1.80**	**(1.24–2.74)**	**1.43**	**(1.01–2.03)**	**1.47**	**(1.04–2.08)**	**1.50**	**(1.06–2.13)**

Models: Model 1: Unadjusted; Model 2: Adjusted for age, sex, household income, educational attainment, economic activity, living arrangement, and alcohol drinking; Model 3: Additionally adjusted for total energy intake and Healthy Eating Index (HEI); Model 4: Model 2 plus total energy intake; Model 5: Model 2 plus HEI.

$p < 0.05$ was considered statistically significant and is shown in **bold**.

Table S4. Sex-stratified associations between TDP and frailty, and tests for interaction

Characteristics	Male		Female		*P* for interaction
Cluster 1	1	(Ref)	1	(Ref)	
Cluster 2	1.52	(0.81-2.85)	1.28	(0.84-1.95)	0.7654
Cluster 3	1.44	(0.79-2.63)	1.25	(0.82-1.89)	0.7744
Cluster 4	**2.05**	**(1.17-3.61)**	1.29	(0.82-2.02)	0.4018
Cluster 5	1.82	(0.98-3.37)	1.32	(0.84-2.08)	0.3561

Models were adjusted for age, sex, household income, educational attainment, economic activity, living arrangement, alcohol consumption, total energy intake and Healthy Eating Index (HEI)

$p < 0.05$ was considered statistically significant and is shown in **bold**.

Table S5. Association between lunch skipping and frailty.

Variable	Model1	Model2	Model3
Lunch Skipping	**1.44 (1.00-2.05)**	1.19 (0.81-1.74)	0.973 (0.66-1.43)

Models: Model 1: Unadjusted; Model 2: Adjusted for age, sex, household income, education, economic activity, living arrangement, and alcohol drinking; Model 3: Additionally adjusted for total energy intake and Healthy Eating Index(HEI).

$p < 0.05$ was considered statistically significant and is shown in **bold**.

Supplementary Figures

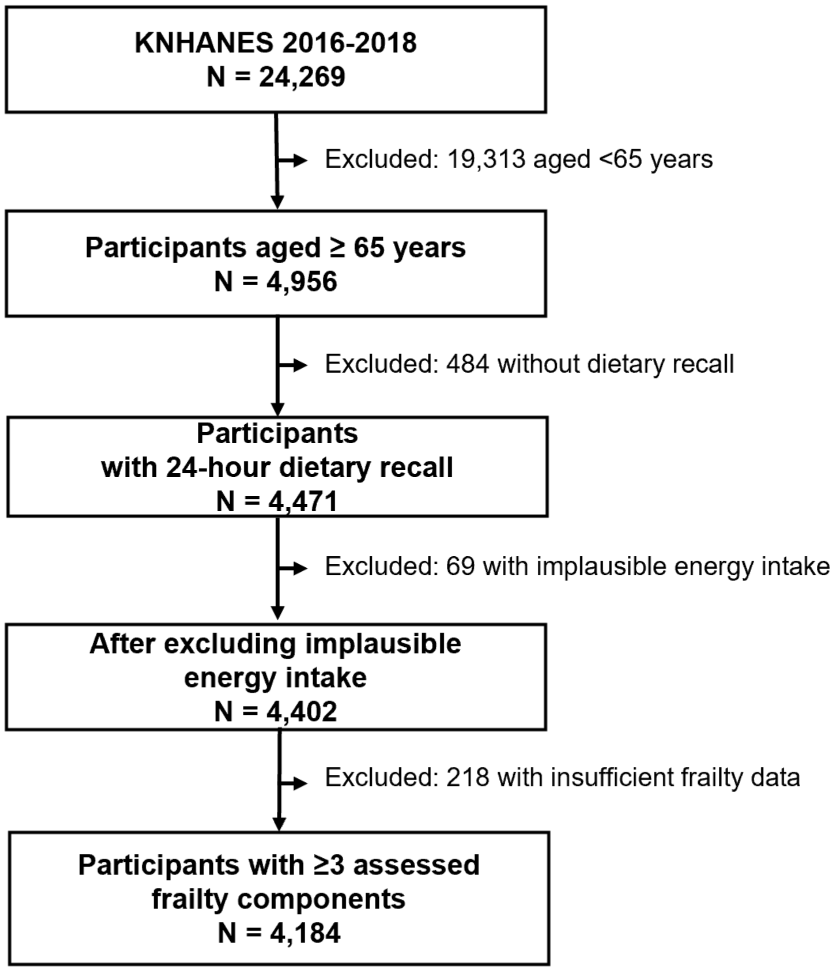

Figure S1. Flow chart of the study design.

KNHANES: Korea National Health and Nutrition Examination Survey

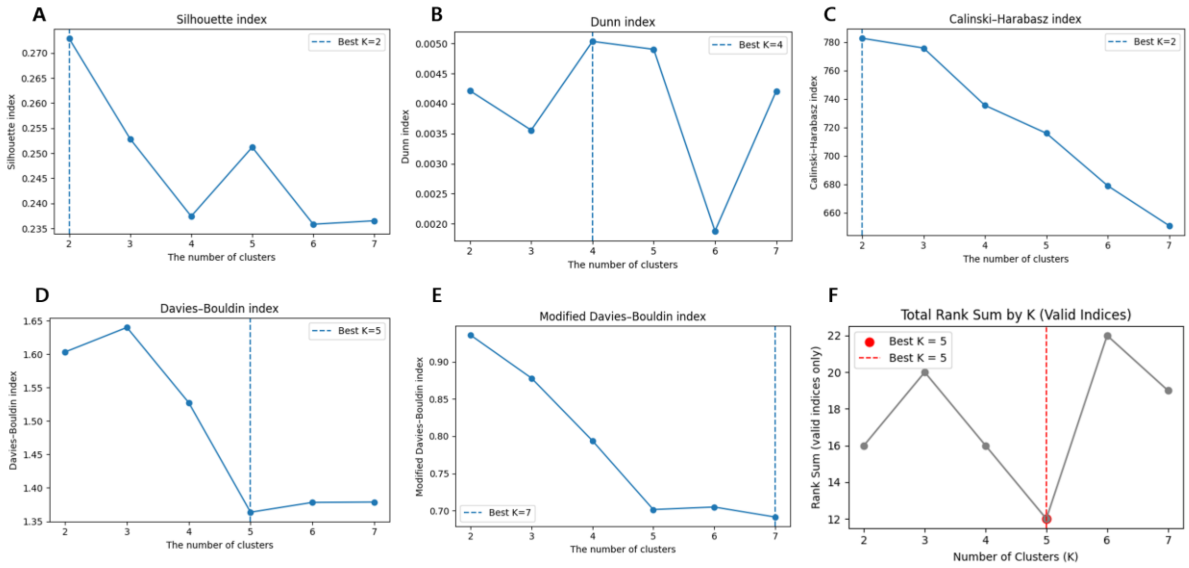

Figure S2. Determination of the optimal number of clusters (K) using a rank-aggregation consensus approach. Multiple internal validity indices were evaluated, including the Silhouette, Dunn, Davies–Bouldin, modified Davies–Bouldin, and Calinski–Harabasz indices. Indices with higher optimal values (**a–c**) and those with lower optimal values (**d–e**) were integrated through the rank aggregation framework (**f**), from which the optimal K was selected.

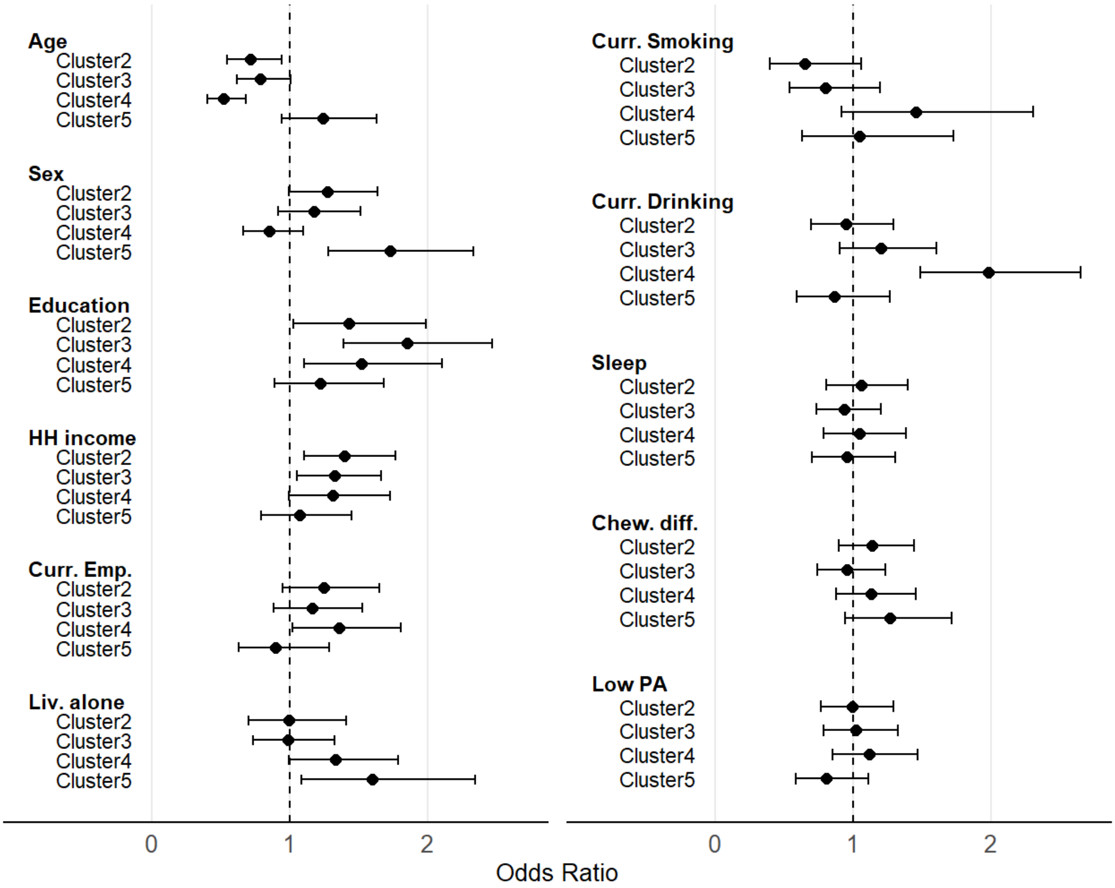

Figures S3. Forest plot showing odds ratios for sociodemographic and health-related characteristics across temporal dietary pattern clusters (reference = Cluster 1). Reference categories for covariates were as follows: age 65–74 years; male sex; education level of middle school or less; lowest household income quartile (Q1); not economically active; living with others; non-smoker; non-drinker; sleep duration ≤6 hours; no chewing difficulty; and moderate- to high-intensity physical activity**.**

Educ.: Education; HH income: Household income; Curr. Emp: Current Employment status; Liv. alone: Living alone; Curr. smoke: Current smoking; Curr. drink: Current drinking; Chew. diff.: Chewing difficulty; Low PA: Low physical activity.

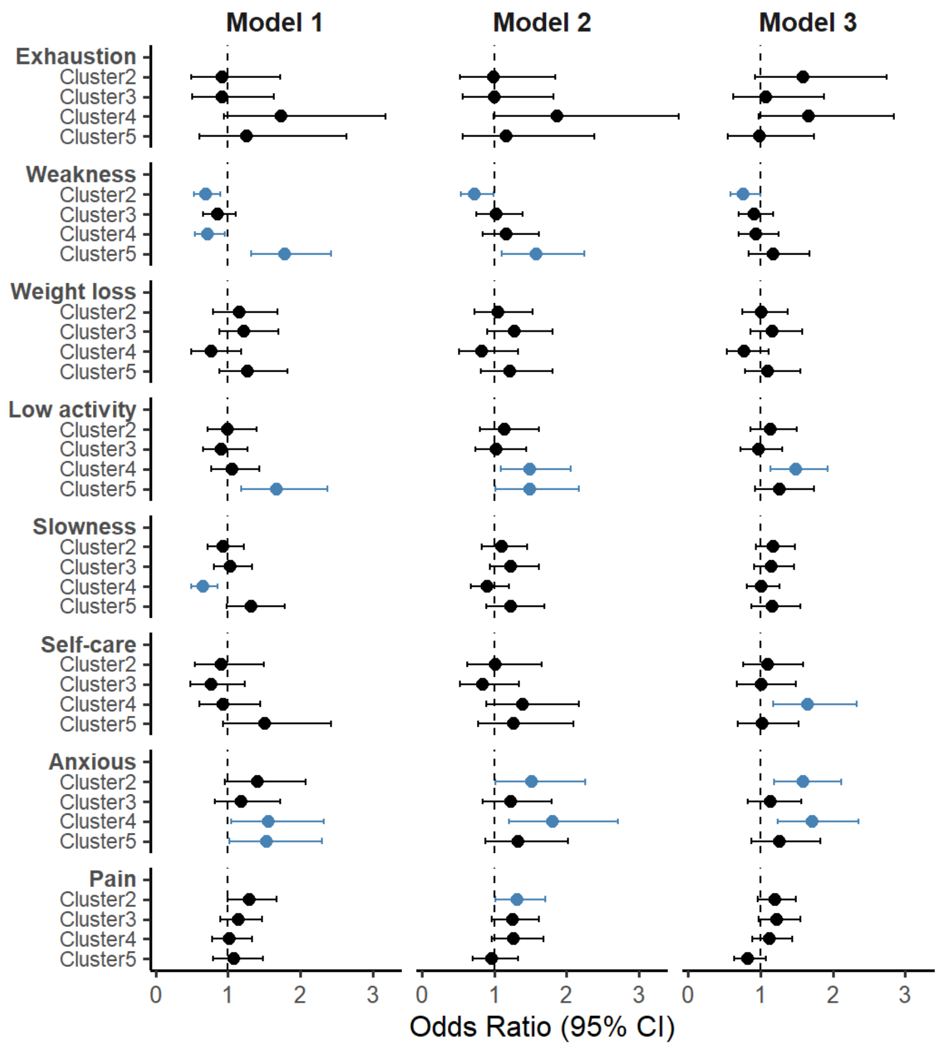

Figure S4. Survey-weighted logistic regression analysis showing odds ratios (ORs) for individual frailty components and EQ-5D dimensions, using Cluster 1 (balanced pattern) as the reference group. All analyses accounted for the complex sampling design of KNHANES, including strata, clusters, and sampling weights. Models 1 and 2 applied combined health interview and examination weights, whereas Model 3 used integrated weights that encompassed the health interview, examination, and nutrition surveys. Model 1: Unadjusted; Model 2: Adjusted for age, sex, household income, educational attainment, economic activity, living arrangement, and alcohol drinking; Model 3: Additionally adjusted for total energy intake and Healthy Eating Index(HEI).

KNHANES: Korea National Health and Nutrition Examination Survey; EQ-5D: EuroQol-5 Dimension questionnaire; CI: confidence interval

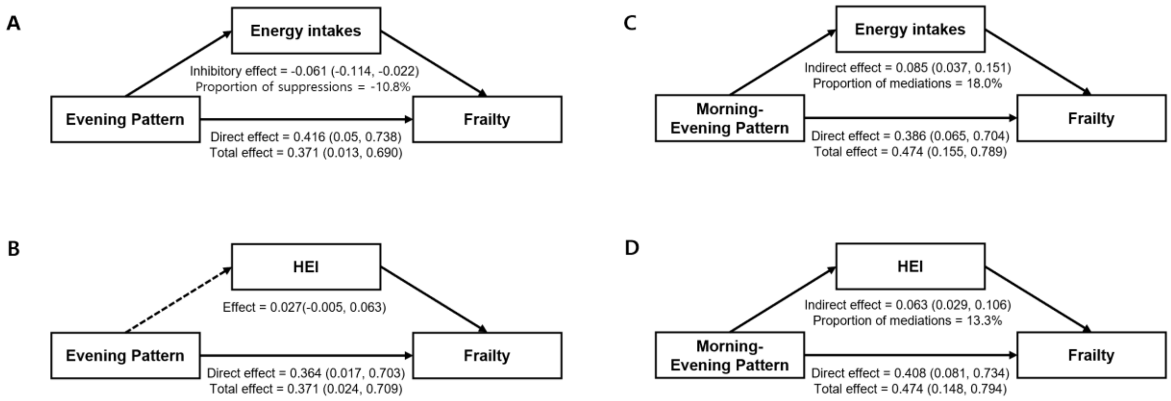

Figure S5. Mediation analysis assessing the indirect and direct effects of temporal dietary patterns on frailty. The evening-skewed pattern was evaluated through (**a**) total energy intake and (**b**) diet quality (Healthy Eating Index, HEI). The morning–evening pattern was evaluated through (**c**) total energy intake and (**d**) HEI.
